# Revisional Therapy for Recurrent Symptoms After Heller Myotomy for Achalasia

**DOI:** 10.1007/s11605-021-05098-8

**Published:** 2021-08-02

**Authors:** Pamela Milito, Stefano Siboni, Andrea Lovece, Erika Andreatta, Emanuele Asti, Luigi Bonavina

**Affiliations:** 1 Department of Biomedical Sciences for Health, Division of General and Foregut Surgery, IRCCS Policlinico San Donato, University of Milan, Milano, Italy; 2 Division of General and Foregut Surgery, IRCCS Policlinico San Donato, Piazza Malan 1, 20097 San Donato Milanese (Milano), Italy

**Keywords:** Achalasia, Recurrent achalasia, Pneumatic dilation, Heller myotomy

## Abstract

**Purpose:**

Symptom recurrence after initial surgical management of esophageal achalasia occurs in 10–25% of patients. The aim of this study was to analyze safety and efficacy of revisional therapy after failed Heller myotomy (HM).

**Methods:**

A retrospective review of a prospective database was performed searching for patients with recurrent symptoms after primary surgical therapy for achalasia. Patients with previously failed HM were considered for the final analysis. The Foregut questionnaire, and the Atkinson and Eckardt scales were used to assess severity of symptoms. Objective investigations routinely included upper gastrointestinal endoscopy and barium swallow study. Redo treatments consisted of endoscopic pneumatic dilation (PD), laparoscopic HM, hybrid Ivor Lewis esophagectomy, or stapled cardioplasty. A yearly clinical and endoscopic follow-up was scheduled in all patients.

**Results:**

Over a 20-year period, 26 patients with a median age of 66 years (IQR 19.5) underwent revisional therapy after failed HM for achalasia at a tertiary-care university hospital. The median time after index procedure was 10 years (IQR 21). Revisional therapy consisted of endoscopic pneumatic dilation (*n*=13), laparoscopic HM and fundoplication (*n*=10), esophagectomy (*n*=2), and stapled cardioplasty and fundoplication (*n*=1). Nine (34.6%) of these patients required further endoscopic or surgical treatments. There was no mortality, and the overall complication rate was 7.7%. At a median follow-up of 42 months (range 10–149), a significant decrease of dysphagia, regurgitation, chest pain, respiratory symptoms, and median Eckardt score (*p*<0.05) was noted.

**Conclusion:**

In specialized and multidisciplinary centers, revisional therapy for achalasia is feasible, safe, and effective.

## Introduction

Achalasia is an esophageal motility disorder characterized by impaired relaxation of the lower esophageal sphincter and loss of esophageal body peristalsis. Available palliative treatments are directed to alleviate symptoms and improve quality of life through reduction of sphincter resistance.^1^ Initial treatment options include surgical Heller myotomy (HM) with anterior Dor fundoplication, endoscopic pneumatic dilation (PD), and, less often, endoscopic injection of botulinum toxin. More recently, per-oral endoscopic myotomy (POEM) has been introduced as a promising therapeutic alternative.^2^

Currently, treatment failure rates are estimated in the range of 20–25% after endoscopic PD and 10–20% after laparoscopic HM.^3^–^7^ Symptom recurrence after PD is related to lack of uniform protocols, operator’s experience, and patient related factors. Recognized causes of surgical failure are an incomplete distal myotomy, twisting or mediastinal herniation of the fundoplication, reflux esophagitis, late fibrosis at the myotomy site, and dolichomegaesophagus. When symptoms persist or recur after the initial therapeutic approach, additional treatment may be required to restore a satisfactory quality of life. The main purpose of this study was to analyze the outcomes of revisional surgery after prior failed Heller myotomy.

## Patients and Methods

Demographic and clinical data of patients diagnosed with achalasia at our tertiary-care referral hospital and esophageal center were retrieved from a prospectively collected electronic database. After internal review board approval, a retrospective study was conducted on all patients undergoing revisional procedures for persistent or recurrent symptoms after primary HM with or without fundoplication. Collected data consisted of demographics, symptoms before index treatment and at baseline, objective endoscopic and radiologic findings, esophageal manometry and pH data if available, intraoperative and postoperative complications, length of hospital stay, and long-term clinical outcomes.

### Pre-operative Work-up

The Foregut questionnaire, the GERD-HRQL questionnaire, and the Atkinson and Eckardt scales were used to assess symptoms severity and frequency. Pre-treatment work-up routinely included upper gastrointestinal endoscopy and barium swallow study. Esophageal manometry and pH-impedance monitoring was performed in selected patients mainly complaining of reflux symptoms. Chest CT scan was performed in elderly individuals to rule out malignancy.

### Revisional Procedures

Depending on age, comorbidities, and results of objective testing, patients were first offered endoscopic PD, laparoscopic HM and Dor fundoplication, laparoscopic transgastric stapled cardioplasty, or hybrid Ivor Lewis esophagectomy. Endoscopic PD was performed after 12 h of fasting, under deep sedation or general anesthesia, depending on patient’s comorbidity and the degree of cooperation, using a 30 or 35 mm Rigiflex™ balloon (Boston Scientific, MA) fully inflated for 60 s, and the balloon inflation was repeated 3 times. Laparoscopic HM was performed after lysis of adhesions between the liver and the phrenoesophageal ligament covering the previous myotomy site which is generally at 12–2 o’clock position; if present, a prior fundoplication was taken down. A mediastinal dissection was performed to enable straightening of a sigmoid esophagus, to reduce a hiatal hernia, or to resect with a linear endostapler an esophageal pseudodiverticulum. Intraoperative endoscopy was selectively used to assist surgical dissection. The HM extended for about 5 cm above the gastroesophageal junction and for 2 cm towards the gastric side, away from the previous myotomy. The most proximal short gastric vessels were divided whenever necessary to relieve tension on the fundic flap, and a 180° anterior fundoplication (Dor) was performed. A posterior crural repair was added whenever necessary, especially when performing a Toupet fundoplication or after reduction of a sigmoid esophagus. Esophagectomy with hybrid Ivor-Lewis approach was reserved to patients with end-stage achalasia and/or multiple prior surgical procedures^8^ in whom a redo HM was considered futile. A laparoscopic trans-gastric stapled cardioplasty^9^ combined with Dor fundoplication was considered in patients with contraindications to esophagectomy. All revisional procedures were carried out by a senior expert surgeon, and the likely reason for symptom persistence/recurrence was annotated in the operative report.

### Follow-up

Clinical and endoscopic follow-up was scheduled at yearly intervals and included symptoms questionnaires and an upper gastrointestinal endoscopy. In patients with persistent/recurrent symptoms during the follow-up, further work-up and revisional treatment was offered depending on patient’s quality of life, previous treatments, and comorbidities. At the latest follow-up, the evolution and change in symptom frequency and severity were analyzed for each patient and compared to baseline.

### Statistical Analysis

Categorical data are reported with frequencies and proportions. Continuous data are reported as median and interquartile range (IQR). Comparison of categorical data was performed using chi-square and Fisher exact tests. Continuous variables were compared using non-parametric tests (Mann–Whitney *U* test, Wilcoxon signed rank test, and Kruskal Wallis test), as appropriate. A *p*-value lower than 0.050 was considered statistically significant. Statistical analysis was performed using Statistical Package for the Social Sciences Software (SPSS 25.0®).

## Results

Between July 1, 2001, and July 31, 2020, among 503 patients treated for achalasia at our institution, 26 (5.2%) presented with persistent/recurrent symptoms after a primary HM. Two (7.7%) of these patients had been previously treated at our center. The median age was 66 years (range 32–87 years), and the median time elapsed between the primary and the revisional procedure was 10 years (range 1–47 years). Among these patients, 22 (84.6%) had a history of trans-abdominal HM, through laparoscopy (*n*=15) or laparotomy (*n*=7), and 4 (15.4%) had trans-thoracic HM. At baseline, dysphagia and regurgitation were the main symptom (96.4% and 80.4%, respectively), followed by chest pain (35.7%), nausea and vomiting (26.8%), and heartburn (10%). The median Eckardt score was 4 (IQR 2.3). The baseline characteristics at presentation and the type of the primary procedures in the patient population are summarized in Table 1.

**Table 1:** Demographic and clinical data of patients who failed prior Heller myotomy (HM) or endoscopic therapy (ET). Values are expressed as median and interquartile range

	**Failed HM** ***n*=26**
**Sex**, M/F	15/11
**Age**, years	66 (19.5)
**BMI**, kg/m^2^	22.6 (3.0)
**Median weight loss,** kg	4.1 ± 5.8
**Comorbidities,** *n* (%)	
Cardiovascular	8 (30.8)
Neurologic	1 (3.8)
Respiratory	4 (15.4)
Metabolic	5 (19.2)
Autoimmune	2 (7.7)
**Median time after index treatment (years)**	10.0 (21)
**Previous procedures**, *n* (%)	
Botox injection	3 (11.5)
Pneumatic Dilation	9 (34.6)
POEM	0 (0.0)
Heller myotomy alone, trans-abdominal	1 (3.8)
Heller myotomy plus fundoplication	21(80.8)
Heller myotomy plus fundoplication, trans-thoracic	4 (15.4)
**Eckardt score**	4.0 (2.3)
**Eckardt grade**	2.5 (1.0)
**Symptoms,** *n* (%)	
Dysphagia	25 (96.2)
Regurgitation	3 (11.5)
Chest pain	20 (76.9)
Nausea/vomiting	4 (15.4)
Heartburn	8 (30.8)
Abdominal pain	6 (23.0)
History of pneumonia	6 (23.0)
**Esophagitis**, grade C, *n* (%)	5 (19.2)
**Resting LES pressure**, mmHg	11.9 (10.0)

### Revisional Procedures

**Figure 1:**
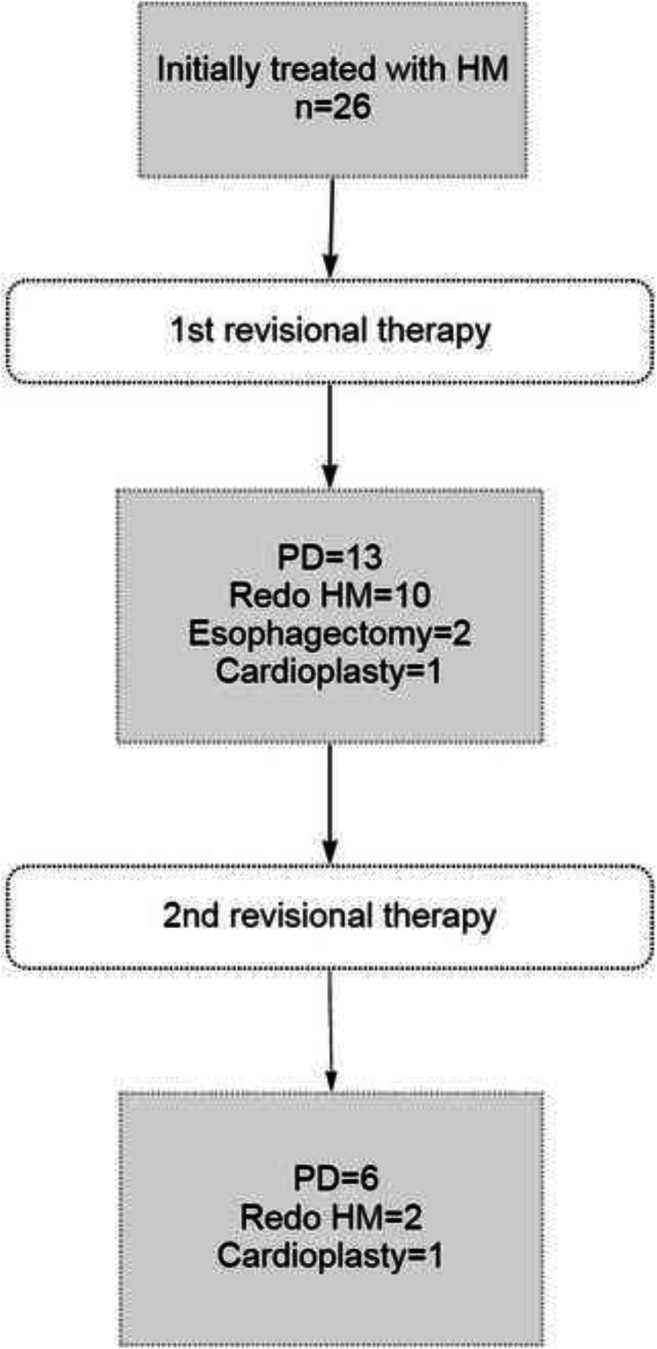
Study flow-chart

**Figure 2:**
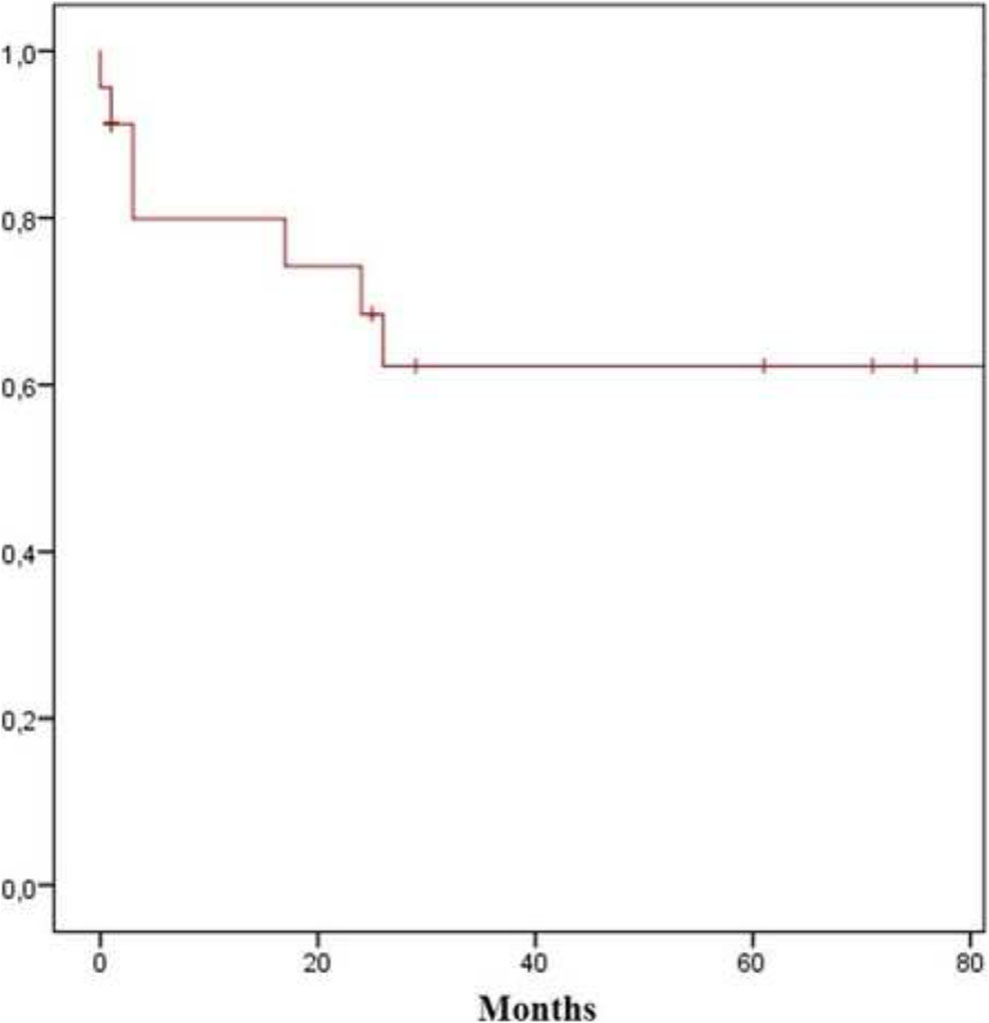
Estimated cumulative probability of success after revisional therapy for achalasia

The most common revisional treatments were PD (50%) and HM with fundoplication (38.5%). There were no conversions to laparotomy. Based on intraoperative assessment, possible reasons for recurrence of symptoms after the primary surgical treatment were the following: dense periesophageal fibrosis (*n*=8), twisted/obstructing fundoplication (*n*=3), dolicho-megaesophagus (*n*=3), disrupted fundoplication (*n*=1), and pseudodiverticulum (*n*=1, 7.7%). One intraoperative mucosal tear occurred during redo HM, and was immediately recognized, repaired, and covered by the Dor flap. Overall postoperative morbidity rate was 7.7% and consisted of atrial fibrillation responsive to pharmacological cardioversion (*n*=1) and left pneumothorax requiring drainage (*n*=1). There was no mortality. The median hospital stay was 3 days (IQR 4). No hospital re-admissions were recorded up to 30 days after discharge (Table 2). Throughout the follow-up time, nine patients (34.6%) required repeat revisional therapy and were treated by PD (*n*=6), redo HM (*n*=2), and stapled cardioplasty (*n*=2) (Figures 1 and 2)

**Table 2:** Treatment modalities, operative time, morbidity, and length of hospital stay

	***n*=26**
**Type of revisional treatment**, *n* (%)	
Pneumatic dilation	13 (50.0)
Heller-Dor	8 (30.8)
Heller-Toupet	2 (7.7)
Esophagectomy	2 (7.7)
Cardioplasty	1 (3.8)
**Associated procedures**, *n* (%)	
Crural repair	5 (19.2)
Diverticulectomy	1 (3.8)
**Median operative time**, min (range)	
Pneumatic dilation	25 (15–30)
Heller myotomy+fundoplication	135 (75–225)
Esophagectomy	392 (305–480)
Cardioplasty	118 (0)
**Complications grade**, *n* (%)	
Clavien-Dindo II	1 (3.8)
Clavien-Dindo IIIb	1 (3.8)
**Median hospital stay**, days, (IQR)	3.0 (4.0)

### Follow up After Revisional Treatments

The median follow-up was 42 months (range 10–149) (Figure 2). There was a statistically significant decrease of dysphagia, regurgitation, chest pain, and respiratory symptoms compared to baseline (*p*<0.05) (Table 3). The overall rate of occasional (once a week or less) dysphagia was 23.0% for the entire cohort. The median Eckardt score significantly decreased from 4.0 (IQR 2.3) to 1.0 (IQR 2.0) (*p*<0.05). At the last endoscopic follow-up, 12 (46.2%) of the 26 patients had normal findings, whereas 5 (19.2%) had a dilated esophagus, 4 (15.4%) a tortuous distal esophagus, 2 (7.7%) grade C distal esophagitis, and 1 (3.8%) a pseudodiverticulum.

**Table 3:** Prevalence and severity of symptoms at baseline and at last follow-up

	***n*=26**		
	**Pre**	**Post**	***p***
**Dysphagia**			<0.001
Absent	1 (3.8)	20 (76.9)	
Occasional	2 (7.7)	6 (23.0)	
Every day	17 (65.4)	0 (0.0)	
Every meal	6 (23.0)	0 (0.0)	
**Regurgitation**			<0.001
Absent	6 (23.0)	25 (96.2)	
Mild	1 (3.8)	1 (3.8)	
Moderate	12 (46.0)	0 (0.0)	
Severe	7 (26.9	0 (0.0)	
**Chest pain**			0.024
Absent	18 (69.2)	25 (96.2)	
Mild	3 (11.5)	1 (3.8)	
Moderate	4 (15.4)	0 (0.0)	
Severe	1 (3.8)	0 (0.0)	
**Respiratory symptoms**	8 (30.8)	0 (0.0)	0.004
**Nausea/vomiting**	6 (23.0)	2 (7.7)	0.249
**Heartburn**			0.609
Absent	23 (88.5)	25 (96.2)	
Occasional	1 (3.8)	1 (3.8)	
Every day	2 (7.7)	0 (0.0)	

## Discussion

The main finding of this study is that revisional therapy after failed HM is safe and effective. Half of our patients were successfully treated by PD at first hand, 38.4% required redo HM, and only 7.7% required esophagectomy. However, one-third of the patients required further endoscopic or surgical treatments. The morbidity rate was low, no major complications occurred, and long-term clinical outcomes were satisfactory.

It has previously been reported^3^ that the presence of adhesions, dense fibrosis, and loss of tissue planes secondary to previous HM cause technical difficulties in approaching the gastroesophageal junction and may decrease the chance of symptom relief, especially after multiple prior interventions and in patients with megaesophagus.^10^ Although laparoscopic surgery after primary HM is considered complex and risky, surgical expertise can minimize conversion rates, provide good clinical outcomes, and allows esophageal preservation.^11^ A recent case-control study comparing primary and revisional laparoscopic HM showed similar clinical and objective (LES integrated residual pressure<15 mmHg) outcomes in both patient groups.^12^

In this study, we attempted to identify the reasons for failure of HM. As classically described by Ellis,^13^ the most frequent causes include an incomplete distal myotomy, sclerosis at the myotomy site, excessively tight fundoplication, reflux esophagitis, or dolichomegaesophagus. Late occurrence of an esophageal pseudodiverticulum, characterized by a blown-out myotomy in the distal esophagus, is an additional cause of persistent bolus retention after HM. This peculiar pattern of failure has recently been defined as >50% increase in esophageal diameter in the area of myotomy above the fundoplication. The Eckardt score and the integrated relaxation pressure are greater in these patients, and type 3 achalasia and HM seem to be the main factors predisposing to a “ballooning” myotomy.^14^ Overall, we found a pseudodiverticulum in 2 (7.7%) patients. It may be speculated that a posterior and long myotomy possibly sparing the longitudinal muscle layer, which is feasible using POEM, may prevent the occurrence of a symptomatic pseudodiverticulum requiring revision and stapled resection.

Because of the wide variety of causes for surgical failure and treatment options, the choice of revisional therapy is challenging, and a standardized approach with a patient-centered multidisciplinary evaluation is highly desirable.^15^ A barium swallow study followed by upper endoscopy represents the preferred initial work-up and it has been suggested that outcomes of reoperation can be predicted based solely on this information.^16^ For the majority of patients, a non-operative approach by PD is a reasonable first choice, and is effective in up to 60% of patients.^17^, ^18^ However, redo HM remains the only viable treatment in patients presenting with mechanical complications such as twisted fundoplication, hiatal hernia, or pseudodiverticulum. Laparoscopic stapled cardioplasty with anterior fundoplication is a feasible and effective first- or second-line revisional procedure in selected patients, but it should be combined with an anterior fundoplication to mitigate postoperative reflux.^19^ Finally, trans-thoracic esophagectomy should be considered the final course of action, and should be reserved to patients with recalcitrant outflow obstruction symptoms and/or preneoplastic mucosal changes after multiple treatment failures.^7^

Per oral endoscopic myotomy is an attractive option to avoid the anterior scar tissue following HM and to perform an extended myotomy along a posterior anatomical plane. In patients with an intact and anatomically competent anterior fundoplication, and no hiatal hernia or pseudodiverticulum, POEM appears an attractive revisional option because it avoids adhesiolysis, is carried out through a virgin posterior plane of dissection opposite to the initial HM, and may cause less reflux since some patients will benefit from the existing Dor fundoplication. Compared to primary POEM, success rate of POEM after HM is lower, complication rates slightly higher, and operative time longer.^2^, ^20^–^26^

### Study Limitations

This is a retrospective study with a relatively small sample size conducted in a single institution, and these results may not be generalizable. In addition, the majority of patients presented after an initial treatment performed elsewhere, and pre- and post-treatment manometric and pH data were not available for all patients. Post-revisional manometric and pH data were not obtained. However, we used the same symptom scales for dysphagia both pre- and postoperatively and the study encompasses a very long period of time, with a median follow-up of 7 years and range from 1 to 15.5 years.

## Conclusion

Revisional endoscopic or surgical treatment for achalasia is feasible, safe, and effective in specialized and multidisciplinary centers, and is associated with minimal postoperative complications and satisfactory long-term outcomes.

## References

[1] Asti E, Sironi A, Lovece A, Bonavina G, Fanelli M, Bonitta G, Bonavina L (2017). Health-related quality of life after laparoscopic Heller myotomy and Dor fundoplication for achalasia. Surgery.

[2] Haisley K, Swanstrom L (2021). The Modern Age of POEM: the Past, Present and Future of Per-Oral Endoscopic Myotomy. J Gastrointest Surg.

[3] Iqbal A, Teirney B, Haider M, Salinas VK, Karu A, Turaga KK, Mittal SK, Filipi CJ (2006). Laparoscopic re-operation for failed Heller myotomy. Dis Esophagus.

[4] Veenstra BR, Goldberg RF, Bowers SP, Thomas N, Hinder RA, Smith CD (2016). Revisional surgery after failed esophagogastric myotomy for achalasia: successful esophageal preservation. Surg Endosc.

[5] Smith KE, Saad AR, Hanna JP, Tran T, Jacobs J, Richter JE, Velanovich V (2020). Revisional Surgery in Patients with Recurrent Dysphagia after Heller Myotomy. J Gastrointest Surg..

[6] Mundre P, Black CJ, Mohammed N, Ford AC (2021). Efficacy of surgical or endoscopic treatment of idiopathic achalasia: a systematic review and network meta-analysis. Lancet Gastroenterol Hepatol..

[7] Patti M, Allaix ME (2015). Recurrent symptoms after Heller myotomy for achalasia: evaluation and treatment. World J Surg.

[8] Aiolfi A, Asti E, Bonitta G, Bonavina L (2018). Esophagectomy for End-Stage Achalasia: Systematic Review and Meta-analysis. World J Surg.

[9] Dehn TCB, Slater M, Trudgill NJ, Safranek PM, Booth MI (2012). Laparoscopic stapled cardioplasty for failed treatment of achalasia. Br J Surg.

[10] Gockel I, Junginger T, Eckardt VF (2007). Persistent and recurrent achalasia after Heller myotomy. Analysis of different patterns and long-term results of reoperation. Arch Surg.

[11] Weche M, Saad AR, Richter JE, Jacobs JJ, Velanovich V (2020). Revisional procedures for recurrent symptoms after Heller myotomy and per-oral endoscopic myotomy. J Laparosc Adv Surg Tech.

[12] Santes O, Coss-Adame E, Valdovinos MA, Furuzawa-Carballeda J, Rodríguez-Garcés A, Peralta-Figueroa J, Narvaez-Chavez S, Olvera-Prado H, Clemente-Gutiérrez U, Torres-Villalobos G. Does laparoscopic reoperation yield symptomatic improvements similar to those of primary laparoscopic Heller myotomy in achalasia patients? Surg Endosc 2020. doi: 10.1007/s00464-020-07978-7. Epub ahead of print. 10.1007/s00464-020-07978-7 32968910

[13] Ellis FH (1997). Failure after esophagomyotomy for esophageal motor disorders: causes, prevention, and management. Chest Surg Clin N Am..

[14] Triggs JR, Krause AJ, Carlson DA, Donnan EN, Campagna RAJ, Jain AS, Kahrilas PJ, Hungness ES, Pandolfino JE (2021). Blown-out myotomy: an adverse event of laparoscopic Heller myotomy and peroral endoscopic myotomy for achalasia. Gastrointest Endosc.

[15] Milito P, Aquilino K, Lazzari V, Boveri S, Munizio N, Ogliari C, Asti E, Bonavina L (2020). The Malnutrition Universal Screening Tool can predict malnutrition in patients with esophageal achalasia. Eur J Gastroenterol Hepatol.

[16] Loviscek MF, Wright AS, Hinojosa MW, Petersen R, Pajitnov D, Oelschlager B, Pellegrini CA (2013). Recurrent dysphagia after Heller myotomy: is esophagectomy always the answer?. J Am Coll Surg.

[17] Zaninotto G, Costantini M, Portale G, Battaglia G, Molena D, Carta A, Costantino M, Nicoletti L, Ancona E (2002). Etiology, diagnosis, and treatment of failures after laparoscopic Heller myotomy for achalasia. Ann Surg.

[18] Saleh CM, Ponds FA, Schijven MP, Smout AJ, Bredenoord AJ (2016). Efficacy of pneumodilation in achalasia after failed Heller myotomy. Neurogastroenterol Motil..

[19] Cosentini EP, Riegler M, Koperek O, Wenzl E (2004). Transgastric stapled esophagofundostomy (TSE) and partial fundoplication – a technical illustration of a new concept for surgical treatment of achalasia. Eur Surg.

[20] Vigneswaran Y, Yetasook AK, Zhao JC, Denham W, Linn JG, Ujiki MB (2014). Peroral endoscopic myotomy (POEM): feasible as reoperation following Heller myotomy. J Gastrointest Surg.

[21] Kristensen HO, Kirkegard J, Kjaer DW, Mortensen FV, Kunda R, Bjerregaard NC (2017). Long-term outcome of peroral endoscopic myotomy for esophageal achalasia in patients with previous Heller myotomy. Surg Endosc.

[22] Louie BE, Schneider AM, Schembre DB, Aye RW (2017). Impact of prior interventions on outcomes during peroral endoscopic myotomy. Surg Endosc.

[23] Tyberg A, Sharaiha RZ, Familiari P, Costamagna G, Casas F, Kumta NA, Barret M, Desai AP, Schnoll-Sussman F, Saxena P, Martínez G, Zamarripa F, Gaidhane M, Bertani H, Draganov PV, Balassone V, Sharata A, Reavis K, Swanstrom L, Invernizzi M, Seewald S, Minami H, Inoue H, Kahaleh M (2018). Peroral endoscopic myotomy as salvation technique post-Heller: International experience. Dig Endosc.

[24] Zanghì S, Toti F, Aiolfi A, Bonavina L (2018). Laparoscopic Heller myotomy and Dor fundoplication after failed POEM: case report and literature review. Eur Surg.

[25] Zhang X, Modayil RJ, Friedel D (2018). Per-oral endoscopic myotomy in patients with or without prior Heller’s myotomy: comparing long-term outcomes in a large U.S. single-center cohort. Gastrointest Endosc.

[26] Modayil RJ, Zhang X, Rothberg B, Kollarus M, Galibov I, Peller H, Taylor S, Brathwaite CE, Halwan B, Grendell JH, Stavropoulos SN. Peroral endoscopic myotomy: 10-year outcomes from a large, single-center U.S. series with high follow-up completion and comprehensive analysis of long-term efficacy, safety, objective GERD, and endoscopic functional luminal assessment. Gastrointest Endosc 2021 [Epub ahead of print] 10.1016/j.gie.2021.05.014 33989646

